# Ruxolitinib early administration reduces acute GVHD after alternative donor hematopoietic stem cell transplantation in acute leukemia

**DOI:** 10.1038/s41598-021-88080-3

**Published:** 2021-04-19

**Authors:** Binglei Zhang, Lingyun Chen, Jian Zhou, Yingling Zu, Ruirui Gui, Zhen Li, Juan Wang, Fengkuan Yu, Yanli Zhang, Huifang Zhao, Zhenyu Ji, Yongping Song

**Affiliations:** 1 Department of Hematology, Affiliated Cancer Hospital of Zhengzhou University and Henan Cancer Hospital, Zhengzhou, 450000 Henan China; 2 Henan Academy of Medical and Pharmaceutical Sciences, Zhengzhou University, Zhengzhou, 450000 Henan China; 3 School of Basic Medical Sciences, Zhengzhou University, Zhengzhou, 450000 Henan China; 4 Academy of Medical Sciences, Zhengzhou University, Zhengzhou, 450000 Henan China

**Keywords:** Leukaemia, Acute lymphocytic leukaemia, Acute myeloid leukaemia

## Abstract

This study aimed to observe the safety and clinical efficacy of early application of ruxolitinib to prevent acute graft-versus-host disease (aGVHD) after alternative donor transplantation in acute leukemia. There were 57 patients undergoing allo-HSCT at the Affiliated Cancer Hospital of Zhengzhou University from July 2017 to October 2019. They were divided into control(16 patients) and ruxolitinib (41 patients) groups. For aGVHD prophylaxis, the control group received post-transplantation cyclophosphamide, antithymocyte globulin-Fresenius, cyclosporine A, and mycophenolate mofetil, while in the ruxolitinib group, ruxolitinib 5 mg/d in adults or 0.07–0.1 mg/(kg d) in children was administered from the day of neutrophil engraftment to 100 days post-transplantation based on control group. We found 55 patients had successful reconstitution of hematopoiesis; No significant difference was found in cGVHD, hemorrhagic cystitis, pulmonary infection, intestinal infection, Epstein-Barr virus infection, cytomegalovirus infection, relapse, death, and nonrelapse mortality. The incidences of aGVHD (50 vs. 22%, *P* = 0.046) and grade II–IV aGVHD (42.9 vs. 12.2%, *P* = 0.013) were significantly higher in the control group than in the ruxolitinib group. No significant differences were observed in overall survival (*P* = 0.514), disease-free survival (*P* = 0.691), and cumulative platelet transfusion within 100 days post-transplantation between two groups. This suggests early application of ruxolitinib can reduce the incidence and severity of aGVHD and patients are well tolerated.

## Introduction

Allogeneic hematopoietic stem cell transplantation (allo-HSCT) is still a potentially curative approach for hematological diseases, especially with the continuous improvement in conditioning regimens and the emergence of new anti-graft-versus-host disease (GVHD) drugs. It has significantly improved the success rate of transplantation. Alternative donors remain an important source. Alternative donor-HSCT has achieved good results; the 4-year overall survival (OS) and disease-free survival (DFS) can be more than 80%^1^. However, transplant-related complications are still pivotal factors decreasing the success of transplantation. Especially acute GVHD (aGVHD)^2,3^is still a common serious complication of allo-HSCT, seriously affecting the survival and prognosis of patients. Many methods are available for preventing and treating GVHD, such as post-transplant cyclophosphamide(PT-Cy)^4^, antithymocyte globulin (ATG)^5^, calcineurin inhibitors (CNIs)^6^, monoclonal antibodies^7^, myeloid-derived suppressor cells^8^, and mesenchymal stem cells^9^. However, efficacy is still not satisfactory. aGVHD remains the main challenge of allo-HSCT. Therefore, new drugs and methods need to be continuously explored to reduce the incidence and severity of aGVHD.

Janus kinase (JAK) is an intracellular nonreceptor tyrosine kinase playing a key role in the development, proliferation, and cytokine signal transduction of various cells (including dendritic cells, macrophages, T cells, B cells, natural killer cells, and neutrophils)^10^. Activated JAKs are necessary for effector T-cell responses in different inflammatory diseases, and their blockade can potentially reduce acute GVHD^11^. Therefore, they are expected to become a new target for the prevention and treatment of GVHD. Ruxolitinib is a selective JAK 1/2 inhibitor, which mainly inhibits the activity of JAK by competitively blocking the binding site of ATP on the catalytic subunit of the JAK1/2 domain; it has the same effect on JAK1 and JAK2. Preclinical studies showed that ruxolitinib has good anti-GVHD effects; it not only has anti-GVHD activity but retains the GVL effect^12^. The Food and Drug Administration approved ruxolitinib for SR-aGVHD in adult and pediatric patients aged 12 years and older on May 24, 2019^13^.

Many clinical studies were performed on the application of ruxolitinib in the treatment of GVHD, confirming that ruxolitinib was effective in steroid-refractory GVHD (SR-GVHD) with few side effects it didn’t increase the risk of recurrence of malignant tumors^14–18^. However, reports on the efficacy of ruxolitinib in preventing GVHD, appropriate dosage, patient tolerance, and impact on survival and prognosis were few. Therefore, the present study mainly retrospectively analyzed the clinical efficacy and safety of the early application of ruxolitinib to prevent aGVHD after transplantation, so as to provide new ideas and directions for the prevention of aGVHD.

### Patients and methods

**Figure 1:**
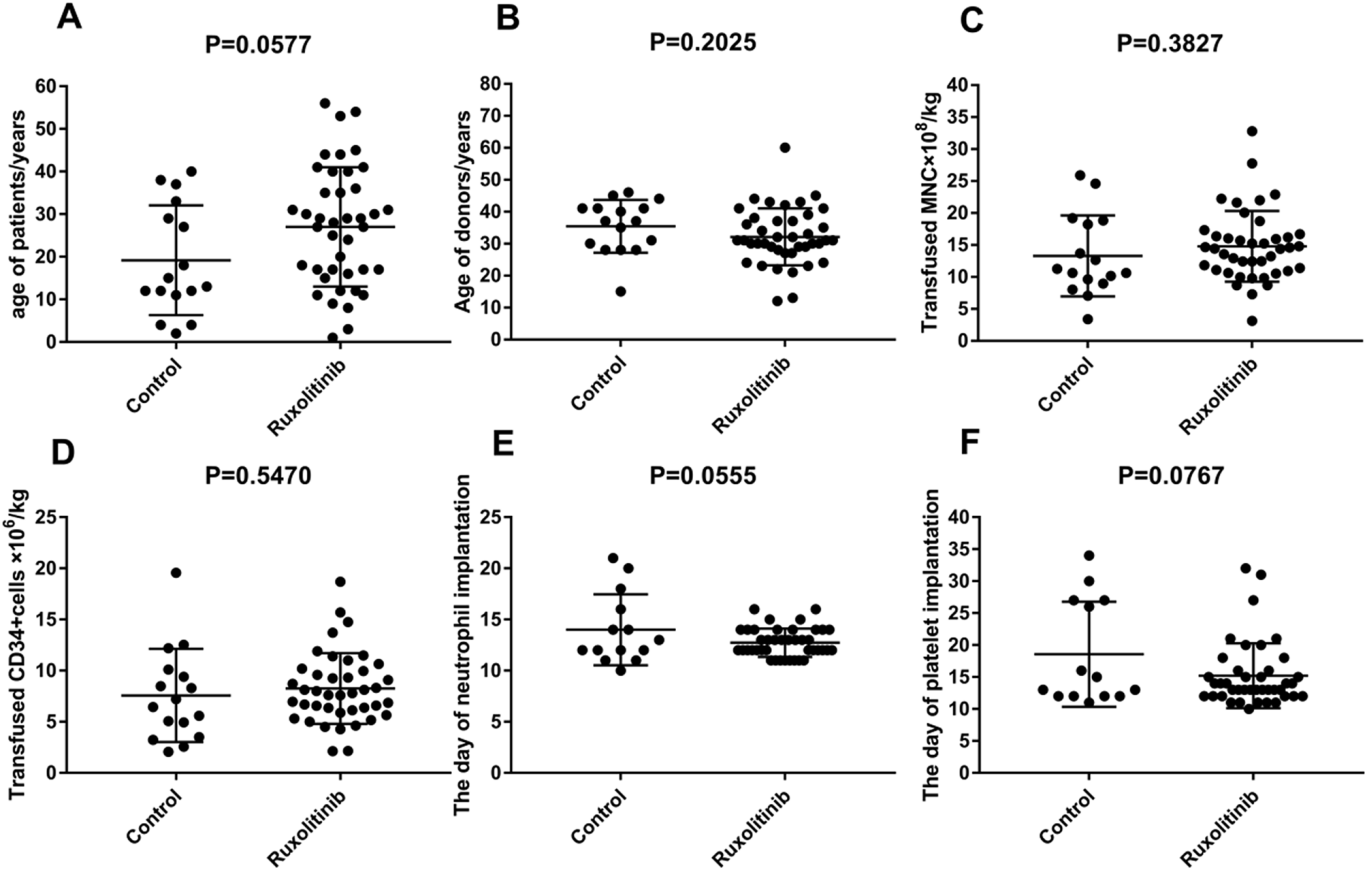
Comparison of characteristics between the control and ruxolitinib groups. Age of patients (**A**), age of donors (**B**), transfused MNC × 10^8^/kg (**C**), transfused CD34 + cells × 10^6^/kg (**D**), day of neutrophil (**E**) and platelet (**F**) engraftment. (Graphpad prism 8.0.1 https://www.graphpad.com/scientific-software/prism/).

The study protocol was approved by the ethics committee of the Affiliated Cancer Hospital of Zhengzhou University. The study was performed in accordance with the Declaration of Helsinki, and all patients themselves or their guardians provided informed consent for their inclusion. The patients underwent allo-HSCT from July 2017 to October 2019 were included in this study. A total of 57 patients were screened (Table 1 and Fig. 1), we comprehensively evaluate the medical condition, the risk of GVHD, financial status and willingness of patients to choose to use ruxolitinib. 16 of whom agreed with common anti-GVHD therapy (control group). 41 of whom agreed with ruxolitinib and common anti-GVHD therapy (ruxolitinib group). Therefore, after allo-HSCT, 57 patients were assigned to control group (N = 16; common anti-GVHD therapy) or ruxolitinib group (N = 41; ruxolitinib and common anti-GVHD therapy).

**Table 1:** Characteristics of all patients in the control and ruxolitinib group (*N* = 57).

	Control (*n* = 16)	Ruxolitinib (*n* = 41)	*χ*^2^	*P* value
Sex (*n*, %)			0.299	0.585
Male	11 (68.8)	25 (61.0)		
Female	5 (31.2)	16 (39.0)		
Primary disease (*n*, %)			0.765	0.382
ALL	5 (31.2)	18 (43.9)		
AML	11 (68.8)	23 (56.1)		
Gene mutation or fusion gene (*n*, %)			1.435	0.231
Yes	10 (62.5)	32 (78.0)		
No	6 (37.5)	9 (22.0)		
Abnormal chromosome (*n*, %)			1.341	0.247
Yes	4 (25.0)	17 (41.5)		
No	12 (75.0)	24 (58.5)		
Disease status at HSCT (*n*, %)			1.93	0.381
CR1	10 (62.5)	32 (78.0)		
CR2 and CR3	3 (18.75)	3 (7.3)		
NR	3 (18.75)	6 (14.7)		
MRD (*n*, %)			0.174	0.677
Positive	6 (37.5)	13 (31.7)		
Negative	10 (62.5)	28 (68.3)		
Sex of donor–recipient (*n*, %)			0.208	0.648
Identical	13 (81.3)	31 (75.6)		
Different	3 (18.7)	10 (24.4)		
Donor–recipient ABO compatibility (*n*, %)			2.557	0.110
Compatible	10 (62.5)	16 (39.0)		
Incompatible	6 (37.5)	25 (61.0)		
Conditioning regimen (*n*, %)			0.043	0.835
TBI/Cy-based	11 (68.7)	27 (65.9)		
Bu/Cy-based	5 (31.3)	14 (34.1)		
Donor source (*n*, %)			2.808	0.246
Haploid donors	10 (62.5)	16 (39.0)		
Matched unrelated donors	5 (31.3)	18 (43.9)		
Mismatched unrelated donors	1 (6.2)	7(17.1)		
Major gene mutation and/or fusion gene (*n*, %)			NA	NA
FLT3/ITD	0 (0)	9 (22.0)		
TET2	6 (37.5)	3(7.3)		
BCR/ABL	2 (12.5)	5 (12.2)		
CEBPA	4 (25.0)	5 (12.2)		
Age	14 (2–40)	28 (1–56)	NA	NA
Age of donors	37 (15–46)	31 (12–60)	NA	NA
MNC × 10^8^/kg	10.6 (3.37–25.88)	14.4 (3.11–32.8)	NA	NA
CD34 + × 10^8^/kg	6.45 (2.07–19.57)	8.02 (2.13–18.69)	NA	NA
Time for engraftment of neutrophils (d)	12 (10–28)	13 (11–20)	NA	NA
Time for engraftment of PLT (d)	13 (11–34)	15 (12–34)	NA	NA

*ALL* Acute lymphoblastic leukemia, *AML* acute myeloid leukemia, *Bu* busulfan, *CR* complete remission, *Cy* cyclophosphamide, *HLA* human leukocyte antigen, *HSCT* hematopoietic stem cell transplantation, *MNC* mononuclear cell, *MRD* minimal residual disease, *NA* not applicable, *NR* no remission, *PLT* platelet, *TBI* total-body irradiation.

For aGVHD prophylaxis, the control group received 20 mg/kg PT-Cy for unrelated donor transplantation and 40 mg/kg for haploid transplantation, + 3 d and + 4 d; antithymocyte globulin-Fresenius (ATG-F), 3.0 mg/(kg ⋅ d), -1 d to -4 d for unrelated donor transplantation, and 5 mg/(kg ⋅ d), + 8 d for haploid transplantation; and cyclosporine A (CsA) and mycophenolate mofetil (MMF), + 5 d. The initial dose of CsA was 2 mg/kg.d for adults and 2.5 mg/kg.d for children. The dose was adjusted according to the concentration of CsA. For haploid transplant patients, the dose will be reduced after 6 months of transplantation and stop for 9–10 months. For unrelated donor transplantation, the dose will be reduced after 6 months of transplantation and stop for 6–8 months. The plasma concentration of CsA was assessed every 3 days and maintained within 150–250 ng/mL. The dosage of MMF usually was 500 mg twice a day, halved at 4 weeks after transplantation, and stopped at 6 weeks, the concentration of MMF was not assessed in all patients for some objective reasons. While in the ruxolitinib group, ruxolitinib 5 mg/d and 0.07–0.1 [mg/(kg ⋅ d)] was administered to adults and children, respectively, from the day of neutrophil engraftment to 100 days post-transplantation based on the control group. In the case of moderate or no aGVHD, ruxolitinib administration was discontinued directly or gradually reduced for severe aGVHD.

In this study, the conditioning regimens included busulfan (Bu)- and cyclophosphamide (CTX)-based regimens (Bu/Cy-based) and total-body irradiation (TBI) combined with CTX-based regimens (TBI/Cy-based) in patients. All patients were given symptomatic and comprehensive support treatment, including the prevention of infection and hemorrhagic cystitis, using granulocyte colony-stimulating factor (G-CSF), and infusion of blood products. In this study, there was no patient received letermovir during the transplant.

The first day when the neutrophil count was more than 0.5 × 10^9^/L for three consecutive days was defined as the neutrophil engraftment time. The first day without platelet transfusion for 7 consecutive days and the platelet count greater than 20 × 10^9^/L was defined as the platelet engraftment time ^19^. After hematopoietic reconstitution, bone marrow specimens were collected and assessed for engraftment by quantitative polymerase chain reaction (Q-PCR) assay or analysis of sex chromosomes. Disease relapse included the hematological and clinical recurrence of leukemia. Nonrelapse mortality was considered as death other than that due to disease relapse. OS was considered the time from the receipt of allo-HSCT to the end of the follow-up or death. DFS was considered from the receipt of allo-HSCT to relapse, death, or end of follow-up. Follow-up was performed via outpatient or inpatient visits, and the follow-up deadline was July 2020.

The classification data were represented as composition ratios. The count data were compared by using the chi-square or Fisher’s exact test, as appropriate. The data of the control and ruxolitinib groups were analyzed using the two-tailed *t* test. The impacts of factors on survival were compared using the log-rank test. Univariate analyses of DFS and OS were performed by the Kaplan–Meier method. The Cox regression model was used for multivariate survival analysis. All statistical analyses were performed using GraphPad Prism (version 8.0.1) and SPSS (version 21.0) software. All statistical tests were two tailed with statistical significance established at *P* < 0.05.

## Results

### Clinical characteristics

The clinical characteristics of patients are summarized in Table 1 and Fig. 1. In this study, all patients received peripheral blood stem cell transplantation in control and ruxolitinib group. Patients received transplantation at the same period, there is no difference in the period of transplantation between two groups. All patients undergo HLA high-resolution testing in both groups. All patients were evaluated for minimal residual disease (MRD) by flow cytometry, and the number of patients with MRD was 6 and 13 in the control and ruxolitinib groups before transplantation, respectively.

The median number of transfused mononuclear cells (MNCs) was 10.6 (3.27–25.88) × 10^8^/kg and 14.4 (3.11–32.8) × 10^8^/kg, and the median number of transfused CD34 + cells was 6.45 (2.07–19.57) × 10^6^/kg and 8.02 (2.13–18.69) × 10^6^/kg in the control and ruxolitinib groups, respectively. No significant differences were found in the basic clinical characteristics between the control and ruxolitinib groups (all *P* > 0.05, Table 1). By the end of follow-up, the rates of cessation of CsA in patients without relapse were 66.7(4/6)% and 72.7(16/22)% in control and ruxolitinib group, respectively.

### Engraftment and complications

**Figure 2:**
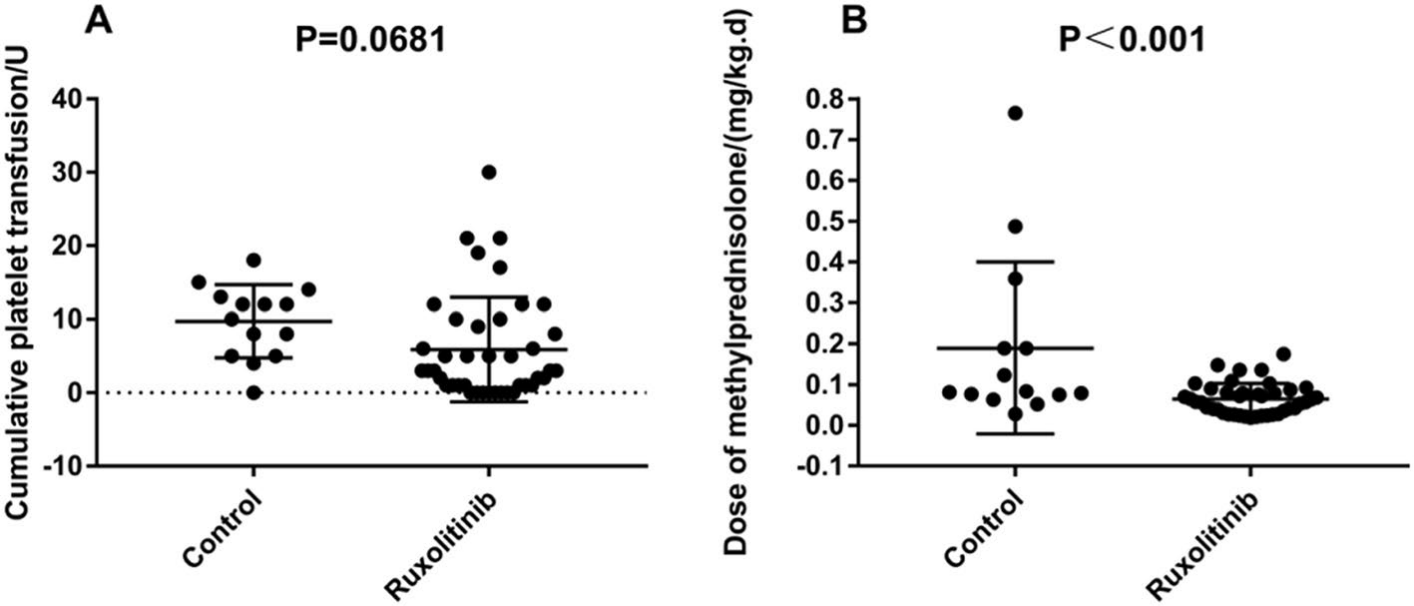
Comparison of the cumulative platelet transfusion (**A**) and the average dose of corticosteroids (converted into methylprednisolone) (**B**) within 100 days after transplantation between the control and ruxolitinib groups. (Graphpad prism 8.0.1 https://www.graphpad.com/scientific-software/prism/).

55 patients had successful reconstitution of hematopoiesis, while 2 patients experienced failure due to early graft rejection and serious infection in the control group. The hematopoietic reconstitution rate was 96.5%. The accumulation time of agranulocytosis did not exceed 7 days within 100 days after neutrophil engraftment in the control and ruxolitinib groups. The median time for neutrophil engraftment was day 12 (range, days 10–28) and day 13 (range, days 11–20), respectively, while the corresponding time for platelet engraftment was day 13 (range, days 11–34) and 15 (range, days 12–34) in the control and ruxolitinib groups, respectively. aGVHD^20^ and chronic GVHD (cGVHD)^21^ were diagnosed and graded by referring to the Seattle standard and the consensus of the National Institutes of Health^22^. No significant differences were observed in the cumulative incidences of cGVHD (*P* = 0.96), hemorrhagic cystitis (*P* = 0.937), pulmonary infection (*P* = 0.783), intestinal infection (*P* = 0.189), Epstein-Barr virus (EBV) infection (*P* = 0.983), and cytomegalovirus (CMV) infection (*P* = 0.967) between the control and ruxolitinib groups. GVHD mainly occurs in the liver, intestine and skin in both groups, there were 5, 5, 3 and 5, 3, 3 patients in the liver, intestine, and skin GVHD in the control and the ruxolitinib group, respectively. The cumulative incidences of aGVHD (50% vs 22% *P* = 0.046) , grade II-IV aGVHD (42.9% vs 12.2%, *P* = 0.013) and grade III-IV aGVHD (28.6% vs 7.3%, *P* = 0.039) were higher for the control than for the ruxolitinib group. The cumulative incidences of cGVHD and severe cGVHD were not significant difference between two groups, but the incidences of severe cGVHD was higher than for the control than for the ruxolitinib group (21.4% vs 12.2%). In addition, 3 and 5 patients were not sensitive to initial steroid therapy in control and ruxolitinib group, respectively. There was not significant difference (*P* = 0.398), and because of the application of ruxolitinib, the average dose of corticosteroids within 100 days for preventing engraftment syndrome and aGVHD after transplantation was significantly higher in the control group than in the ruxolitinib group (*P* < 0.001), while the cumulative platelet transfusion within 100 days between the control and ruxolitinib groups was not significantly different (*P* = 0.0681). (Table 2 and Fig. 2).

**Table 2:** Complications and prognosis of all patients in the control and ruxolitinib groups (*N* = 55).

	Control (*n* = 14)	Ruxolitinib (*n* = 41)	*χ*^2^	*P* value
aGVHD (*n*, %)			3.980	0.046
Yes	7 (50.0)	9 (22.0)		
No	7 (50.0)	32 (78.0)		
Grade II–IV aGVHD (*n*, %)			6.132	0.013
Yes	6 (42.9)	5 (12.2)		
No	8 (57.1)	36 (87.8)		
Grade III-IV aGVHD (*n*, %)			4.245	0.039
Yes	4 (28.6)	3 (7.3)		
No	10 (71.4)	38 (92.7)		
cGVHD (*n*, %)			0.002	0.960
Yes	4 (28.6)	12 (29.3)		
No	10 (71.4)	29 (70.7)		
Severe cGVHD (*n*, %)			0.716	0.398
Yes	3 (21.4)	5 (12.2)		
No	11 (78.6)	36 (87.8)		
Hemorrhagic cystitis (*n*, %)			0.006	0.937
Yes	7 (50.0)	21 (51.2)		
No	7 (50.0)	20 (48.8)		
Pulmonary infection (*n*, %)			0.076	0.783
Yes	9 (64.3)	28 (68.3)		
No	5 (35.7)	13 (31.7)		
Intestinal infection (*n*, %)			1.725	0.189
Yes	6 (42.9)	10 (24.4)		
No	8 (57.1)	31 (75.6)		
EBV infection (*n*, %)			0.000	0.983
Yes	1 (7.1)	3 (7.3)		
No	13 (92.9)	38 (92.7)		
CMV infection (*n*, %)			0.002	0.967
Yes	11 (78.6)	32 (78.0)		
No	3 (21.4)	9 (22.0)		
Relapse (*n*, %)			0.352	0.553
Yes	3 (21.4)	6 (14.6)		
No	11 (78.6)	35 (85.4)		
Death (*n*, %)			0.487	0.485
Yes	8 (57.1)	19 (46.3)		
No	6 (42.9)	22 (53.7)		
Nonrelapse mortality (*n*, %)			0.317	0.574
Yes	6 (42.9)	16 (39.0)		
No	2 (14.3)	3 (7.3)		

*CMV* Cytomegalovirus, *EBV* Epstein-Barr virus, *GVHD* graft-versus-host disease.

### Prognosis in the control and ruxolitinib groups

After a median follow-up of 9.5 (range 1.4–31.5) months, 3 and 6 patients experienced disease relapse in the control and ruxolitinib groups, respectively. The recurrence rate was not significantly different in the control and ruxolitinib group (21.4% vs 14.6%, *P* = 0.553). Further, 8 and 19 patients died in the control and ruxolitinib groups, respectively. Thus, the mortality rate (57.1% vs 46.3%, *P* = 0.485) and nonrelapse mortality (NRM) rate (42.9% vs 39%, *P* = 0.574) were not statistically significantly different in the control and ruxolitinib groups (Table 2). The main causes of non-relapse-related deaths of the patients were organ failure and severe pulmonary infection.

### Survival analyses in 55 patients

**Figure 3:**
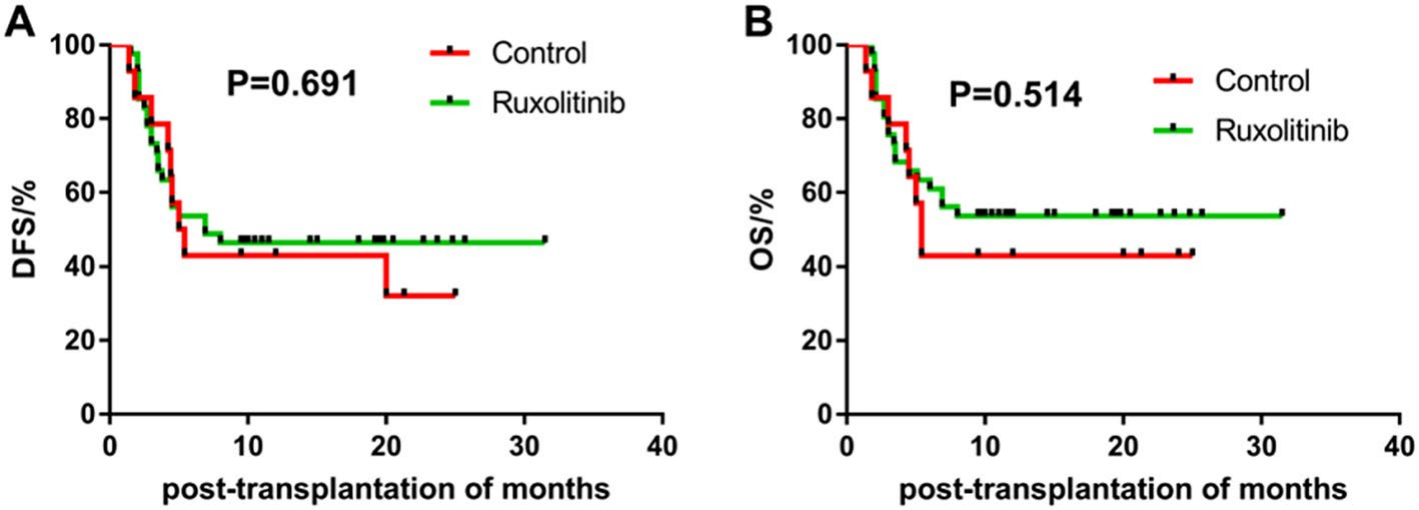
DFS (**A**) and OS (**B**) rates of 55 patients between the control and ruxolitinib groups. (Graphpad prism 8.0.1 https://www.graphpad.com/scientific-software/prism/).

The survival analysis showed no significant difference in OS (*P* = 0.514) and DFS (*P* = 0.691) between the control and ruxolitinib groups. The 2-year OS was (42.9 ± 13.2)% and (53.7 ± 7.8)%, and the 2-year DFS was (32.1 ± 13.6)% and (46.3 ± 7.8)%, respectively, in the control and ruxolitinib groups (Table 3 and Fig. 3 ). In addition, univariate and multivariate survival analyses were also performed on 55 patients, the results were showed in Table3 and Supplementary file.

**Table 3:** Univariate survival analysis of OS and DFS after allo-HSCT $\left( {\overline{x} \pm s} \right)$ (*N* = 55).

Variables	No. of patients	OS		DFS	*P*
		Rate/%	*P* value	Rate/%	*P* value
Age			0.719		0.549
≤ 25	27	14.007 ± 2.052		12.263 ± 2.044	
> 25	28	18.621 ± 2.625		16.713 ± 2.596	
Sex			0.906		0.591
Male	34	17.679 ± 2.380		16.791 ± 2.388	
Female	21	15.2 ± 2.418		12.248 ± 2.297	
Primary disease			0.187		0.296
ALL	23	20.761 ± 2.804		18.291 ± 2.888	
AML	32	12.988 ± 1.886		11.918 ± 1.829	
Gene mutation and/or fusion gene			0.872		0.891
Yes	41	18.087 ± 2.164		15.929 ± 2.16	
No	14	14.571 ± 2.985		13.98 ± 2.886	
Abnormal chromosome			0.984		0.45
Yes	21	14.681 ± 2.369		11.695 ± 2.286	
No	34	17.774 ± 2.366		16.922 ± 2.363	
Disease status at HSCT			0.433		0.392
CR1	41	18.756 ± 2.151		17.366 ± 2.163	
CR2 and CR3	6	8.767 ± 3.407		8.767 ± 3.407	
NR	8	13.813 ± 3.613		10.044 ± 3.128	
MRD			0.030		0.005
Positive	18	10.194 ± 2.318		8.069 ± 1.971	
Negative	37	20.422 ± 2.215		18.957 ± 2.246	
Sex of donor–recipient			0.585		0.649
Identical	42	17.467 ± 2.172		16.697 ± 2.177	
Different	13	13.777 ± 2.284		9.885 ± 2.145	
Donor–recipient ABO compatibility			0.277		0.209
Compatible	25	12.976 ± 2.148		11.272 ± 2.077	
Incompatible	30	19.607 ± 2.492		17.694 ± 2.517	
Conditioning regimen			0.406		0.872
TBI/Cy-based	36	14.072 ± 1.846		13.386 ± 1.849	
Bu/Cy-based	19	19.942 ± 3.119		15.622 ± 3.074	
Donor source			0.298		0.545
Haploid donors	25	13.048 ± 2.137		12.192 ± 2.11	
Unrelated donors	30	19.547 ± 2.504		16.765 ± 2.515	
HLA matching			0.541		0.707
HLA-match donors	23	16.048 ± 2.303		14.388 ± 2.286	
HLA-mismatched donors	32	16.863 ± 2.443		15.1 ± 2.41	
GVHD prophylaxis			0.514		0.691
Control	14	12.914 ± 2.813		12.3 ± 2.705	
Ruxolitinib	41	18.641 ± 2.17		16.554 ± 2.178	
aGVHD			0.003		0.008
Yes	16	8.675 ± 2.377		8.258 ± 2.225	
No	39	21.008 ± 2.133		18.785 ± 2.207	
Grade II–IV aGVHD			0.002		0.003
Yes	11	7.173 ± 2.575		6.718 ± 2.312	
No	44	20.277 ± 2.040		18.307 ± 2.088	
cGVHD			0.003		< 0.001
Yes	16	26.9 ± 2.396		26.806 ± 2.448	
No	39	11.967 ± 1.748		9.877 ± 1.608	
Hemorrhagic cystitis			0.356		0.992
Yes	28	15.886 ± 2.569		15.789 ± 2.585	
No	27	16.537 ± 2.134		13.521 ± 2.111	
Pulmonary infection			0.105		0.445
Yes	37	15.7 ± 2.278		14.795 ± 2.262	
No	18	18.533 ± 2.401		14.839 ± 2.573	
Intestinal infection			0.703		0.41
Yes	16	13.125 ± 2.436		10.2 ± 2.046	
No	39	18.503 ± 2.252		17.056 ± 2.258	
EBV infection			0.282		0.205
Yes	4	13.25 ± 1.516		13.25 ± 1.516	
No	51	17.286 ± 1.958		15.09 ± 1.926	
CMV infection			0.279		0.193
Yes	43	15.986 ± 2.114		14.621 ± 2.09	
No	12	19.1 ± 2.468		16.369 ± 2.598

## Discussion

Acute GVHD is a serious complication of allo-HSCT and an important factor threatening the survival of patients. The incidence of aGVHD can also be as high as 30%–50% with a classic prophylaxis regimen^23^. The prevention and treatment of GVHD is still an important challenge of allo-HSCT. Ruxolitinib can impair the differentiation of CD4^+^ T cells into IFN-γ- and IL17A-producing cells, and both T-cell phenotypes are linked to GVHD. Thus, ruxolitinib may be a novel targeted drug in GVHD by inhibiting proinflammatory signaling that mediates tissue damage^11^. In recent years, ruxolitinib achieved significant efficacy as a very promising drug for the treatment of GVHD, with an overall response rate of up to 100%^24^. The 2-year OS was 61.2%^25^. In a retrospective multicenter survey from 19 centers, the overall response rate of ruxolitinib reached 81.5% and 85.4% in steroid-refractory aGVHD and cGVHD, respectively^26^. However, reports on the use of ruxolitinib in the prevention of GVHD are few^27^.

In the present study, the clinical efficacy, adverse effects, and incidences of other transplant-related complications in the control and ruxolitinib groups were compared. The incidences of aGVHD (22% vs 50%, *P* = 0.046), grade II–IV aGVHD (12.2% vs 42.9%, *P* = 0.013) and grade III–IV aGVHD (7.3% vs 28.6%, *P* = 0.039) were significantly lower in the ruxolitinib group than in the control group. Zhao et al.also suggested that ruxolitinib was applied at 5–10 mg twice daily until 2–3 months after transplantation, or reduced gradually with GVHD and discontinued by 6 months. The prophylactic application of ruxolitinib after allo-HSCT seemed to be safe and effective for preventing GVHD^28^. Additionally, the incidence of aGVHD after transplantation was significantly higher in the control group than in the ruxolitinib group (*P* < 0.001). This may also be related to the fact that the control group has more haploid and mismatched unrelated donors, although it is not statistically significant (*P* = 0.246). The major complication and side effects of ruxolitinib were CMV reactivation and cytopenia^26^. However, in this study, the cumulative platelet transfusion within 100 days after transplantation (*P* = 0.0681) and other transplant-related complications in the control and ruxolitinib groups were not significantly different. While, because of the application of ruxolitinib, the dose of steroids was significantly reduced. The accumulation time of agranulocytosis did not exceed 7 days within 100 days after neutrophil engraftment in the control and ruxolitinib groups. The amount of red blood cell transfusion within 100 days after transplantation was not statistically analyzed in the control and ruxolitinib groups, because patients might have hemorrhagic cystitis or gastrointestinal bleeding; this needs further exploration. These results indicated that the early application of ruxolitinib to prevent aGVHD reduced the incidence and severity of aGVHD. Also, patients had good tolerance, and the incidences of adverse effects of hematopoiesis and other transplant-related complications did not increase.

In addition, survival analysis was performed on 55 patients with hematopoietic reconstruction. OS (*P* = 0.514) and DFS (*P* = 0.691) were not significantly different in the control and ruxolitinib groups; the 2-year OS was (42.9 ± 13.2)% and (53.7 ± 7.8)%, and the 2-year DFS was (32.1 ± 13.6)% and (46.3 ± 7.8)%, respectively. The survival rate was lower than that in other reports, which might be related to the existence of poor prognosis gene mutations and abnormal chromosomes in patients. Poor gene mutations made patients insensitive to chemotherapy, leading to high-dose chemotherapy damages to the function of multiple organs, and various transplant-related complications such as infection and organ failure appeared in the early stage of transplantation, which affected the NRM, OS and DFS to a certain extent. However, ruxolitinib seemed to improve patients' OS and DFS to some extent. In this study, the main cause of NRM of the patients was non-GVHD-related death. The main causes of non-GVHD-related deaths of the patients were organ failure and severe pulmonary infection. There was no increase in mortality due to the application of ruxolitinib, which also shows the safety of ruxolitinib. This was similar to preventing GVHD in myelofibrosis after allo-HSCT^29^, indicating that the early application of ruxolitinib for preventing GVHD did not negatively influence the outcome after allo-HSCT. However, infection remained an important factor leading to nonrelapse mortality of patients, which needed attention. In addition, univariate and multivariate survival analyses were also performed on 55 patients, the results are consistent with previous research reports^19,30–33^. This further shows that the application of ruxolitinib does not affect the survival of patients.

Although ruxolitinib had a significant effect on the prevention and treatment of GVHD, some problems still needed to be resolved. First, the perfect time to apply ruxolitinib to prevent GVHD: are neutrophils implanted, or both neutrophils and platelets implanted? Second, the perfect time to discontinue ruxolitinib: can we quickly discontinue other immunosuppressants instead of stopping ruxolitinib to reduce the incidence of infection without significant cytopenia after engraftment? These issues need to be addressed to make better use of ruxolitinib.

In summary, the early application of ruxolitinib reduced the incidence and severity of aGVHD. The patients were well tolerated, and the incidence of other transplant-related complications did not increase and affect the survival and prognosis of patients. Of course, the sample size of our study is not yet sufficient, and the findings would be validated by a large mutli-center randomized clinical trials.

## Supplementary Information

Supplementary Information 1.

Supplementary Information 2.

Supplementary Legends.
