# Supplementary figures and images for: Ruxolitinib early administration reduces acute GVHD after alternative donor hematopoietic stem cell transplantation in acute leukemia
Source: Sci Rep. 2021 Apr 19;11:8501. doi: 10.1038/s41598-021-88080-3 (PMC8055912; doi:10.1038/s41598-021-88080-3)

Supplementary Figure

Figure1


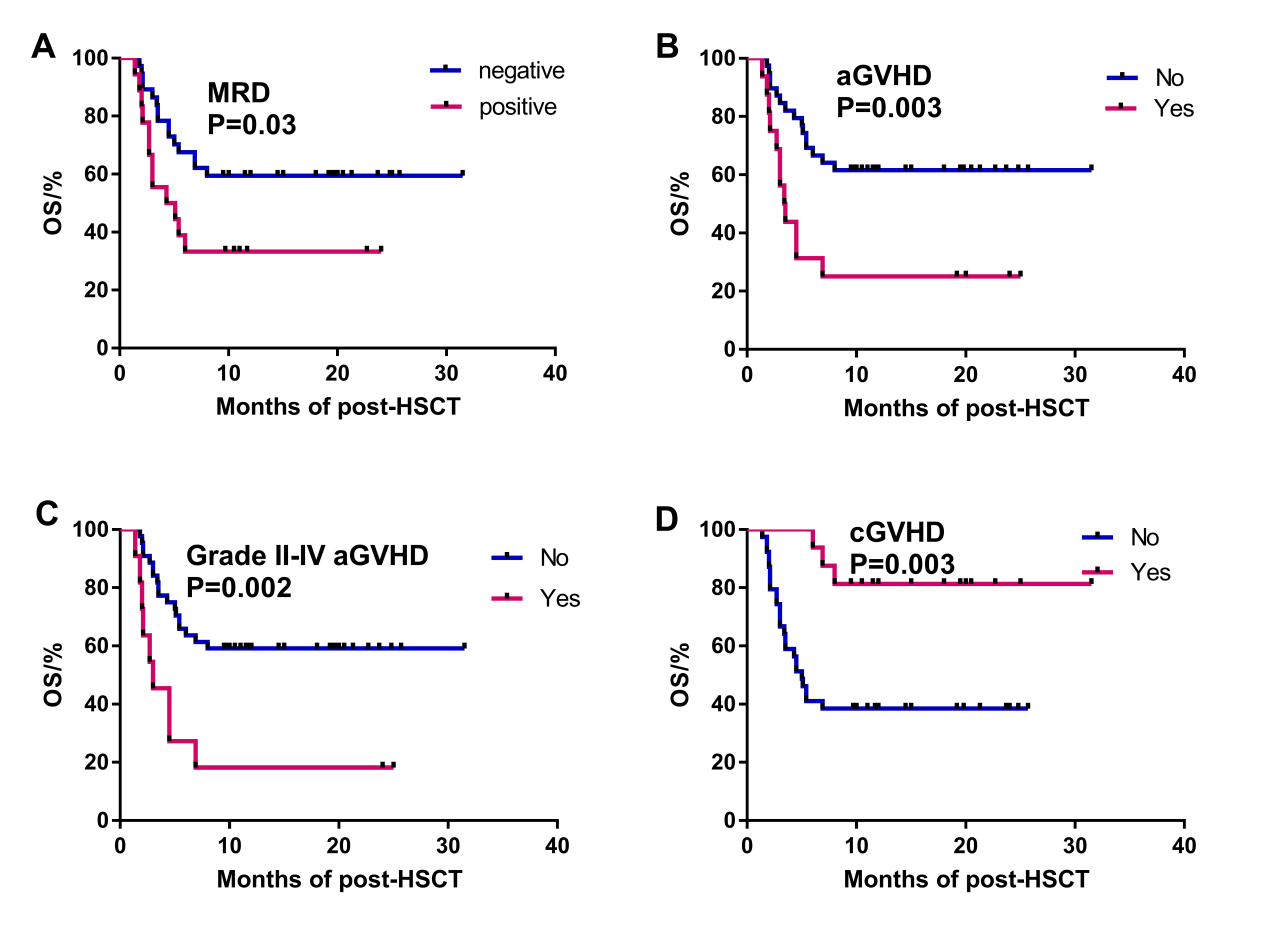


Figure2


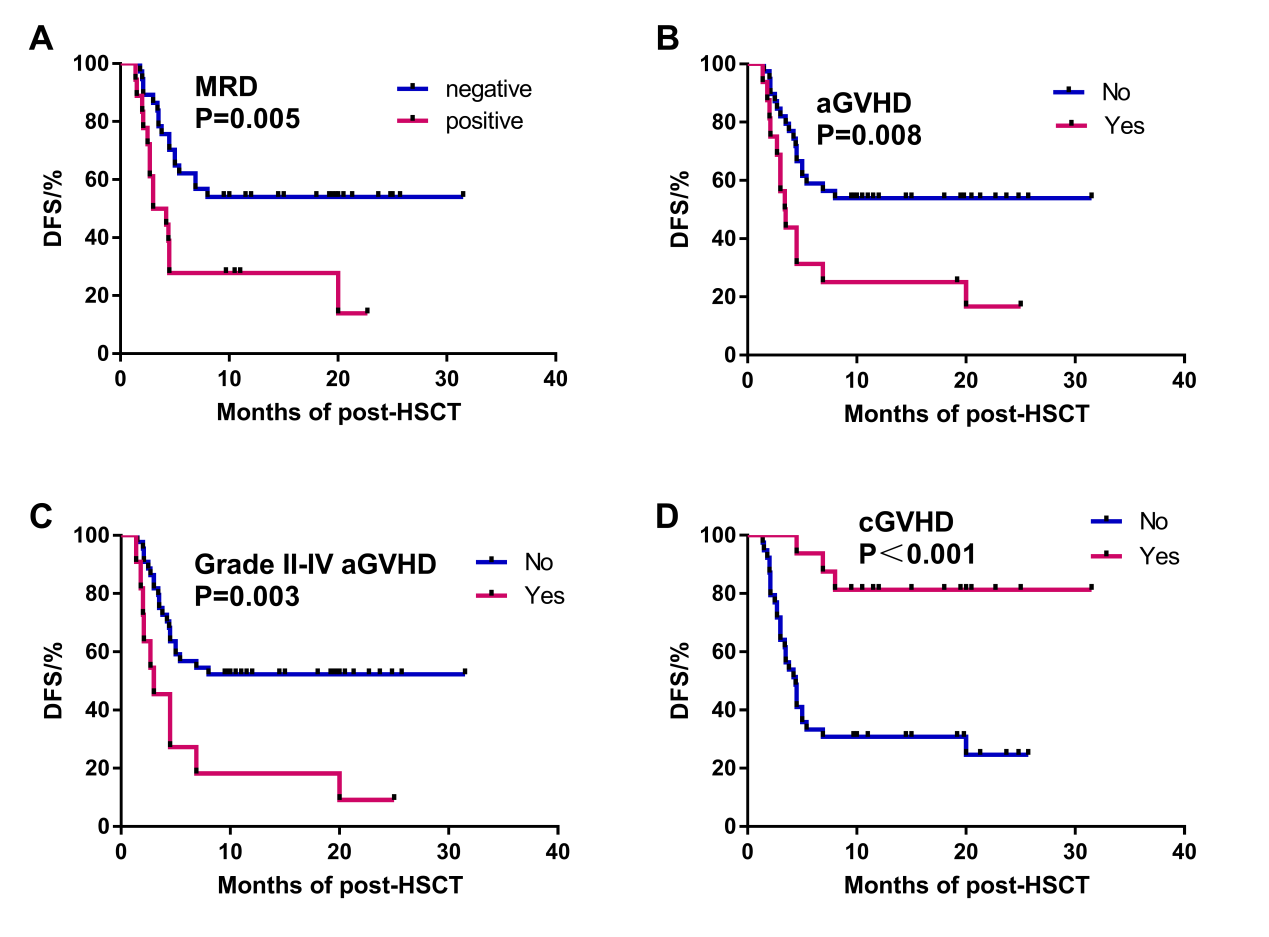

Supplement: Supplementary file 1 — Supplementary Information 1. [file 41598_2021_88080_MOESM1_ESM.docx]
