# Supplementary material for: Ruxolitinib early administration reduces acute GVHD after alternative donor hematopoietic stem cell transplantation in acute leukemia
Source: Sci Rep. 2021 Apr 19;11:8501. doi: 10.1038/s41598-021-88080-3 (PMC8055912; doi:10.1038/s41598-021-88080-3)
Supplement: Supplementary file 2 — Supplementary Information 2. [file 41598_2021_88080_MOESM2_ESM.docx]

Supplementary Table

Table 1. Multivariate survival analysis of OS after allo-HSCT

|  | *B* | SE | Wald | df | Sig. | Exp(B) | 95.0% CI for Exp(B) | |
| --- | --- | --- | --- | --- | --- | --- | --- | --- |
|  |  |  |  |  |  |  | Lower | Upper |
| MRD | –.574 | .404 | 2.022 | 1 | .155 | .563 | .255 | 1.243 |
| aGVHD | .677 | .646 | 1.097 | 1 | .295 | 1.967 | .555 | 6.977 |
| Grade II–IV aGVHD | .325 | .685 | .226 | 1 | .635 | 1.385 | .361 | 5.304 |
| cGVHD | –1.385 | .628 | 4.872 | 1 | .027 | .250 | .073 | .856 |

Table 2. Multivariate survival analysis of DFS after allo-HSCT

|  | *B* | SE | Wald | df | Sig. | Exp(B) | 95.0% CI for Exp(B) | |
| --- | --- | --- | --- | --- | --- | --- | --- | --- |
|  |  |  |  |  |  |  | Lower | Upper |
| MRD | –.735 | .378 | 3.785 | 1 | .052 | .479 | .229 | 1.005 |
| aGVHD | .449 | .634 | .502 | 1 | .479 | 1.567 | .452 | 5.428 |
| Grade II–IV aGVHD | .270 | .678 | .158 | 1 | .691 | 1.310 | .347 | 4.949 |
| cGVHD | –1.659 | .620 | 7.152 | 1 | .007 | .190 | .056 | .642 |
