# Supplementary material for: Ruxolitinib early administration reduces acute GVHD after alternative donor hematopoietic stem cell transplantation in acute leukemia
Source: Sci Rep. 2021 Apr 19;11:8501. doi: 10.1038/s41598-021-88080-3 (PMC8055912; doi:10.1038/s41598-021-88080-3)
Supplement: Supplementary file 3 — Supplementary Legends. [file 41598_2021_88080_MOESM3_ESM.docx]

Figure1 Impacts of status of MRD (A), aGVHD (B), grade II–IV aGVHD (C), and cGVHD (D) on OS in 55 patients.

Figure 2. Impacts of the status of MRD (A), aGVHD (B), grade II–IV aGVHD (C), and cGVHD (D) on DFS in 55 patients.
